# The burden, distribution and risk factors for cervical oncogenic human papilloma virus infection in HIV positive Nigerian women

**DOI:** 10.1186/1743-422X-11-5

**Published:** 2014-01-15

**Authors:** Oliver Chukwujekwu Ezechi, Per Olof Ostergren, Francisca Obiageri Nwaokorie, Innocent Achaya Otobo Ujah, Karen Odberg Pettersson

**Affiliations:** 1 Clinical Sciences Division, Nigerian Institute of Medical Research, Lagos, Nigeria; 2 Division of Social Medicine and Global Health, Faculty of Medicine, Lund University, Lund, Sweden; 3 Molecular Biology Division, Nigerian Institute of Medical Research, Lagos, Nigeria

**Keywords:** Human papilloma virus (HPV), Cervical cancer, HIV, Antiretroviral therapy

## Abstract

**Background:**

The expected reduction in cervical cancer incidence as a result of increased access to antiretroviral therapy is yet to be seen. In this study we investigated the effect of HIV infection and treatment on high-risk (hr) human papilloma virus (HPV) prevalence and distribution.

**Methods:**

Cervical cells from 515 (220 HIV positive and 295 HIV negative) women, recruited during community cervical cancer screening programme in states of Ogun and Lagos and at the cervical cancer screen clinic, Nigerian Institute of Medical Research Lagos were evaluated for the presence of 13 hr HPV genotypes by polymerase chain reaction based assay.

**Results:**

The prevalence of high-risk HPV was 19.6% in the studied population. HPV 16 (3.9%), 35 (3.5%), 58 (3.3%) and 31 (3.3%) were the most common hr HPV infections detected. We observed that the prevalence of hr HPV was higher in HIV positives (24.5%) than 15.9% in HIV negative women (OR = 1.7; 95% CI: 1.1-2.7). A multivariate logistic regression analysis showed a lower hr HPV prevalence in HIV positive women on antiretroviral drugs (OR = 0.4; 95% CI: 0.3-0.5) and with CD4 count of 500 and above (OR = 0.7; 95% CI: 0.5-0.8). A higher prevalence of hr HPV was also noted in HIV positive women with CD4 count < 200 cells/mm3 (OR = 2.4; 95% CI: 1.7-5.9).

**Conclusion:**

HPV 16, 35, 58 and 31 genotypes were the most common hr HPV infection in our study group, which could be regarded as high risk general population sample; with higher prevalence of HPV 16 and 35 in HIV positive women than in HIV negative women. The use of antiretroviral drugs was found to be associated with a lower prevalence of hr HPV infection, compared to those not on treatment. This study raises important issues that should be further investigated to enable the development of robust cervical cancer prevention and control strategies for women in our setting.

## Background

In sub-Saharan Africa, the cervical cancer rates are on the rise, paralleling the HIV epidemic [1,2]. The observed rise in cervical cancer rate has been linked to the chronic deviations in the immune systems due to HIV infection [3-5]. The impaired cell mediated immunity due to HIV infection results in the body’s inability to clear high risk (hr) human papilloma virus (HPV) leading to the persistence of the virus in the cervix and eventual transformation of the infected cervical cells to precancerous and cancerous lesions [6]. With the diverse HIV epidemic in sub-Saharan Africa, it is essential to consider how it may affect HPV related cervical diseases including cervical cancer [7,8].

While studies from southern and eastern Africa have noted a strong association between HIV, HPV co-infection and the development of genital cancers [2,3,9,10], we were unable to identify studies from the West African sub-region that reported on HIV and HPV infection interactions. Considering the HIV viral diversity across regions and the differential effect of the viral strains on disease progression, it is expected that there may be regional variations in the reported association between HIV, HPV co-infection and development of cervical cancer [11,12].

The introduction of antiretroviral therapy in the last few years and the increased access to HIV care in low-income countries have resulted in the improvement of clinical outcomes and life expectancy in HIV infected persons [7,13,14]. Consequently, similar improvement was projected in cervical cancer related morbidity and mortality [7,15,16]. However, decades after the introduction of antiretroviral drugs, the expected reduction in new cervical cancer cases and deaths is yet to be seen [16]. This obvious absence of an association between the projected improvement in cervical cancer outcomes and reported cases globally has triggered investigations on the interaction between HIV, HPV and cervical cancer [17-19].

Epidemiological studies, including meta-analyses mostly from high-income countries, have shown that HIV infected women are at increased risk of infection with hr HPV, yet studies from sub-Saharan Africa that accounts for over 70% of global cervical cancer burden contribute less than 1% of the evidence [2,9,19-21]. Prevention and control strategies based on these data sets, might not be effective in a sub-Saharan African context especially not in West Africa where limited information on HPV and HIV co-infection is available.

The two currently available vaccines that protect against the acquisition of hr HPV are based on HPV 16 and 18, the reported most common HPV genotypes globally [9,22-24]. However, there is increasing evidence of regional and sub-regional variations in HPV genotype distribution, suggesting that the current vaccines may not be as effective in the sub-Saharan Africa region as projected [2,7,21,23-25]. Previous studies from sub-Saharan Africa show that although the burden of HPV is high compared to Europe and North America, a lower prevalence of HPV 16 and 18 and a higher prevalence of other hr HPV genotypes such as HPV 31, 35 and 58, were observed [22-28]. A plausible assumption could therefore be that other non-HPV 16 and 18 oncogenic genotypes may account for thousands of new cases of cervical cancer that occur in this region annually. In addition, since immunity to HPV virus like particle vaccines is type-specific, it is critical to characterize the distribution of hr HPV genotypes in the West African sub region with high burden of both HIV and HPV infection, in order to develop an effective vaccine targeting women in this region [2,7,22,23,28,29].

Unfortunately, several low-income countries, including Nigeria, have approved and deployed the HPV vaccine without such a detailed characterization of the HPV genotypes in their settings [30]. Considering the amount of resources invested in procuring and distributing this vaccine, it is imperative to conduct studies that will determine the prevalence of prevailing HPV genotypes in these countries. Such information will potentially contribute to the development of second-generation HPV vaccines, which will improve prevention and control of cervical cancer in the sub-region [2,3].

Increasingly in Nigeria and other West African countries, a large number of women have been offered an opportunity to HIV treatment [13,31]. It is therefore important to determine the impact of such long-term treatment on the HPV prevalence and distribution as well as on cervical cancer, in order to develop effective contextual cervical cancer prevention for HIV positive women.

Since the introduction of HIV treatment in Nigeria in 2002, over 350,000 HIV infected adults have been initiated on antiretroviral therapy [30] and 60% of these are women [18]. Unfortunately, little or no information exists on the prevalence, and distribution of HPV and cervical cancer in this cohort [15,18,31]. In addition, extensive literature search shows no study that has evaluated the effect of HIV infection, immunosuppression and antiretroviral drug use on the burden and distribution of HPV infection.

This study therefore aims to determine the hr HPV prevalence and distribution by HIV status among women residing in two southwestern Nigerian states of Lagos and Ogun. The effect of HIV infection, immunosuppression and antiretroviral drug use on the hr HPV distribution was also assessed.

## Results

A total of 536 (231 HIV positive and 305 HIV negative) women were enrolled into the study over a period of 12 months. The HPV results of 21 (3.9%) women were invalid and thus excluded from the analysis. There were no differences in demographic profiles of these 21 women and the remaining 515 (96.1%) with valid HPV results (p = 0.8). Table 1 shows the sociodemographic characteristics of the study participants by recruitment site. Although the women recruited from NIMR site were slightly younger and more likely to have a secondary education, the observed differences were not statistically significant. Only 6.5% of the women recruited from the community were living with HIV compared to 80.6% among the NIMR site cohorts (p = 0.00).

**Table 1:** Comparison of the sociodemographic characteristics of the women by recruitment sites

**Characteristics of participants**	**NIMR site**	**Community site**	**P value**
	**n = 262 (%)**	**n = 253 (%)**	
**Median age (years)**	35 [IQR: 31-41]	37 [IQR:32–47]	0.09
**Educational level completed**			
< Secondary	53 (21.0)	69 (26.2)	0.20
≥ Secondary	199 (79.0)	194 (73.8)	
**Marital status**			
Unmarried	88 (34.9)	85 (32.3)	0.60
Married	164 (65.1)	178 (67.7)	
**Tribal group**			
Major Southern tribes	151 (59.9)	159 (60.4)	0.57
Northern tribes	54 (21.4)	48 (18.3)	
Southern minority tribes	47 (18.7)	56 (21.3)	
**Residence**			
Urban	90 (35.7)	93 (35.4)	0.99
Rural	162 (64.3)	170 (64.6)	
**Occupation**			
Unemployed	34 (13.5)	38 (14.4)	0.85
Employed	218 (86.5)	225 (85.6)	
**Age at first intercourse (years)**			
< 15	27 (10.7)	30 (11.4)	0.91
≥ 15	225 (89.3)	223 (88.6)	
**Number of life time sexual partners**			
< 2	91 (36.1)	83 (31.6)	0.31
≥ 2	161 (63.9)	180 (68.4)	
**HIV status**			
Negative	49 (19.4)	246 (93.5)	0.00
Positive	203 (80.6)	17 (6.5)	

### Socio-demographic characteristics

The socio-demographic characteristics of 515 women with valid HPV results by HIV status is shown in Table 2. Of these women with valid results, 332 (64.5%) and 183(35.5%) resided in rural and urban communities respectively. The age of the women ranged from 18 – 81 years with a median age of 37 years [IOR: 31–45]. The majority (56.0%) were aged between 20 and 39 years. Over sixty percent (60.4%) of the women belonged to the two main ethic groups in southern Nigeria – Yoruba (33.3%) and Igbo (27.1%), with the northern ethnic groups constituting 19.7%. Approximately 67% were married and the remaining were either single (16.7%), divorced (5.2%) or widowed (11.7%). A majority of the women had at least a secondary education (76.3%), were engaged in an income generating activity (86.0%) and had at least one previous delivery (77.1%). The age of the women at their first intercourse ranged from 9–38 years with a mean of 20.4 ± 3.9 years. Over two-thirds of the women in the study reported total lifetime sexual partner between 2 and 10 with a mean of 2.9 ± 2.5.

**Table 2:** Socio-demographic characteristics of the 515 women by hr HPV infection status

**Characteristics**	**Number of women (%)**	**hr HPV infection status**		**OR; 95% Confidence interval (CI)**
	**n = 515**	**hr HPV positive status**	**hr HPV negative status**	
		**n = 101 (%)**	**n = 414 (%)**	
**Age (years)**				
< 25	51 (9.9)	19 (37.3)	32 (62.7)	2.2 (1.1-4.6)
25 – 34	165 (32.0)	35 (26.9)	130 (73.1)	1 (ref)
≥ 35	299 (58.1)	47 (18.7)	252 (81.3)	0.7 (0.4-1.2)
**Educational level completed**				
< Secondary	122 (13.7)	23 (18.9)	99 (81.1)	0.9 (0.5-1.6)
≥ Secondary	393 (76.3)	78 (19.8)	315 (80.2)	1 (ref)
**Marital status**				
Never married	86 (16.7)	24 (27.9)	62 (72.1)	1.9 (1.1-3.4)
Married	342 (66.4)	58 (16.9)	284 (83.1)	1 (ref)
Divorced/Separated/Widow	87 (16.9)	19 (21.8)	68 (78.2)	1.4 (0.7-2.5)
**Tribal group**				
Major Southern tribes	310 (60.2)	57 (18.4)	253 (81.6)	1 (ref)
Northern tribes	102 (19.8)	21 (20.6)	81 (79.4)	1.2 (0.6-2.1)
Southern minority tribes	103 (20.0)	23 (22.3)	80 (77.7)	1.3 (0.7-2.3)
**Residence**				
Urban	183 (35.5)	44 (24.0)	139 (76.0)	1.6 (1.0-2.6)
Rural	332 (64.5)	54 (16.3)	278 (83.7)	1 (ref)
**Occupation**				
Unemployed	72 (14.0)	20 (27.8)	52 (72.2)	1.7 (0.9-3.1)
Employed	443 (86.0)	81 (18.2)	362 (81.8)	1 (ref)
**Age at first intercourse (years)**				
< 15	57 (11.1)	13 (22.8)	44 (77.2)	1.3 (0.6-2.6)
15 – 24	378 (73.3)	72 (19.0)	306 (81.0)	1 (ref)
≥ 25	80 (15.5)	16 (20.0)	64 (80.0)	1.1 (0.6-2.0)
**Number of life time sexual partners**				
1	174 (33.8)	23 (13.2)	151 (86.8)	1 (ref)
2 – 4	256 (49.7)	57 (22.3)	199 (77.7)	1.9 (1.1-3.3)
≥ 5	85 (16.5)	21 (24.7)	64 (75.3)	2.2 (1.1-4.4)
**Number of deliveries**				
0	118 (22.9)	21 (17.8)	97 (82.2)	0.9 (0.5-1.6)
1 – 4	244 (47.4)	48 (19.7)	196 (80.3)	1 (ref)
≥ 5	153 (29.7)	32 (20.9)	121 (79.1)	1.1 (0.6-1.8)
**HIV status**				
Negative	295 (57.3)	47 (15.9)	248 (84.1)	1 (ref)
Positive	220 (42.7)	54 (24.5)	166 (75.5)	1.7 (1.1-2.7)

### Laboratory and treatment characteristics of the HIV positive women

The HIV related characteristics of HIV positive women enrolled in the study is presented in Table 3. The CD4 cell count of the women ranged from 10 to 1651 cells/mm 3 with a median of 500 [IQR: 347–685]. The majority (50.9%) of the women had CD4 cells counts above 500 cells/mm 3. The plasma viral load levels ranged from undetectable levels (<20) to 657,899 copies/ml with a median of 200 [IQR: 200–2270]. Over 60% had viral loads less than 1000 copies/ml. While the majority (72.3%) were already on antiretroviral therapy (ART) at enrolment for a period ranging from 3 months to 8 years, others were yet to start ART (27.7%).

**Table 3:** Laboratory and treatment characteristics of the 220 HIV positive women by hr HPV infection status

**Variables**	**Number of women n = 220 (%)**	**hr HPV infection status**		**OR; 95% confidence interval (CI)**
		**hr HPV positive status**	**hr HPV negative status**	
		**n = 101 (%)**	**n = 414 (%)**	
**CD4 cell count (Cells/mm3)**				
< 200	22 (10.0)	12 (54.5)	10 (45.5)	3.1 (1.1-9.1)
200 – 499	86 (39.1)	24 (27.9)	62 (72.1)	1 (ref)
>500	112 (50.9)	18 (16.1)	94 (83.9)	0.5 (0.2-1.0)
**HIV viral load (Copies/ml)**				
<1000	136 (61.8)	19 (14.0)	117 (86.0)	0.5 (0.2-1.8)
1000 – 9999	26 (121.8)	6 (23.1)	20 (76.9)	1 (ref)
>10,000	58 (26.4)	29 (50.0)	29 (50.0)	3.3 (1.1-10.9)
**Antiretroviral drug use**				
Not on drugs	61 (27.7)	24 (39.3)	37 (60.7)	1 (ref)
On drugs	159 (72.3)	30 (18.9)	129 (81.1)	0.4 (0.2-0.7)

### High-risk HPV genotype distribution

Table 4 shows the distribution of hr HPV strains by HIV status, the Odds Ratios and 95% CI, after adjustment for age, antiretroviral treatment and life time sexual partnership. The hr HPV prevalence in the study was 19.6% and multiple hr HPV infections were detected in 5.4% of the women. The most commonly detected hr HPV genotypes were 16 (3.9%), 35 (3.5%), 58 (3.3%) and 31 (3.3%), followed by 18 (2.3%), 52 (2.3%) and 51 (1.9%). HPV genotypes 16 and 18 infections were detected in 32 (21.7%) of the 101 hr HPV infected women in this study.

**Table 4:** The distribution of hr HPV, overall and by specific genotypes among the women by HIV status

**hr HPV status**	**All women n = 515 (%)**	**HIV positive n = 220 (%)**	**HIV negative n = 295 (%)**	**OR (95% CI)**	
				**Crude**	**Adjusted**^**a**^
HR HPV negative	414 (80.4)	166 (75.4)	248 (84.1)	1 (ref)	1 (ref)
HR HPV positive	101 (19.6)	54 (24.6)	47 (15.9)	1.9 (1.2–2.9)	1.7 (1.1-3.1)
HPV 16	20 (3.9)	14 (5.4)	6 (2.6)	3.3 (1.1-9.7)	3.1 (1.3-6.3)
HPV 18	12 (2.3)	3 (1.4)	9 (3.10	0.4 (0.1-1.8)	0.4 (0.2-1.9)
HPV 31	17 (3.3)	12 (5.5)	5 (1.7)	3.1 (1.1-11.1)	1.9 (0.9-8.1)
HPV 33	2 (0.4)	1 (0.5)	1 (0.3)	1.3 (0.0-49.3	1.1 (0.0-46.1)
HPV 35	18 (3.5)	13 (5.9)	5 (1.7)	3.4 (1.2-11.9)	3.6 (1.4-6.9)
HPV 39	3 (0.6)	1 (0.5)	2 (0.7)	0.7 (0.1-9.4)	0.6 (0.2-9.1)
HPV 45	7 (1.4)	3 (1.4)	4 (1.4)	1.0 (0.2-5.4)	0.9 (0.1-5.9)
HPV 51	10 (1.9)	5 (2.3)	5 (1.7)	1.4 (0.3-5.4	1.4 (0.4-5.5)
HPV 52	12 (2.3)	6 (2.7)	6 (2.0)	1.4 (0.4-4.8)	1.3 (0.2-5.1)
HPV 56	6 (1.2)	2 (0.9)	4 (1.4)	0.7 (0.1-4.3)	0.7 (0.2-6.9)
HPV 58	18 (3.5)	9 (4.1)	9 (3.5)	1.4 (0.5-3.9)	1.2 (0.3-4.1)
HPV 59	3 (0.6)	1 (0.5)	2 (0.7)	0.7 (0.02-9.4)	0.5 (0.1-10.1)
HPV 68	4 (0.8)	1 (0.5)	3 (1.0)	0.4 (0.02-4.8)	0.4 (0.1-6.1)
Multiple HPV infection	28 (5.4)	18 (8.2)	10 (3.9)	2.5 (1.1-6.1)	2.6 (1.3-5.3)

^a^adjusted for confounders of age, antiretroviral drug use and number of life time sexual partners.

The prevalence of hr HPV was higher in HIV positive (24.5%) women than in HIV negative women (15.9%; OR = 1.7; 95% CI: 1.1-2.7). Multiple infection rates were also higher in the HIV positive women (8.2%) compared to 3.9% in HIV negative women (OR = 2.6; 95% CI: 1.3-5.3). A similar trend was observed in HPV 16 (OR = 3.1; 95% CI: 1.3-6.3) and HPV 35 (OR = 3.6; 95% CI: 1.4-6.9) infections. No difference was observed in other 11 hr HPV genotypes studied.

Among the HIV positive women, HPV 16 and 18 constituted 32.7% [18] of hr HPV infections compared to 30.6% [14] among HIV negative women, which was not statistically significant (p = 0.99).

### Association between HIV infection, treatment and hr HPV infection

To determine the association between HIV infection, treatment and hr HPV infection, we compared the sociodemographic and HIV related characteristics with hr HPV status among the women in the study (Tables 2, 3 and 5). Differences in the status of hr HPV infection were found for various variables first at univariate analysis and then multivariate analysis for variables found significant at univariate analysis. At multivariate model to avoid over or under correction for confounding variables, the data were analyzed and reported in three models (Table 5).

**Table 5:** Variables independently associated with hr HPV after controlling for confounders in a three model multivariate logistic analysis

**Variables at enrolment**	**Initial model**^**a**^	**2nd Model**^**b**^	**Final model**^**c**^
	**OR (95% CI)**	**OR (95% CI)**	**OR (95% CI)**
**HIV status (515)**			
Negative	1 (ref)	1 (ref)	1 (ref)
Positive	1.7 (1.3 -2.5)	1.7 (1.3-2.5)	1.8 (1.4-2.2)
**Antiretroviral drug use (220)**			
Not on drugs	1 (ref)	1 (ref)	1 (ref)
On drugs	0.4 (0.3 - 0.9)	0.4 (0.3 - 0.9)	0.4 (0.3-0.5)
**CD4 cell count (220)**			
<200	2.9 (1.3-7.1)	2.5 (1.5-6.1)	2.4 (1.7-5.9)
200 – 499	1 (ref)	1 (ref)	1 (ref)
500 and above	0.6 (0.3-1.0)	0.6 (0.5-0.9)	0.7 (0.5-0.8)
**Viral load (220)**			
<1000	0.5 (0.2-1.9)	0.6 (0.4 -1.3)	0.6 (0.3 -1.4)
1000 – 10,000	1 (ref)	1 (ref)	1 (ref)
≥ 10,000	3.1 (1.1-9.9)	3.1 ( 0.9- 8.7)	2.7 (0.6- 8.1)

^a^adjusted for age ^b^adjust for HIV status and antiretroviral drug use ^c^in addition to a and c adjusted for type of community, life time sexual partner and marital status.

At univariate analysis (Tables 2 and 3), a greater percentage of women with hr HPV infection status were less than 25 years (OR: 2.2; 95% CI: 1.1 – 4.6), have never married (OR: 1.9; 95% CI: 1.1-3.4), resides in an urban community (OR: 1.6; 95% CI: 1.0-2.6), and have had at least two life time sexual partners (OR: 1.9; 95% CI: 1.1-1.3.3). HIV positive women (OR: 1.7; 95% CI: 1.1 – 2.7), those with CD4 cell count less than 200 (OR: 3.1; 95% CI: 1.1-9.1) and viral load greater than 10,000 copies (OR: 3.3; 95% CI: 1.0-10.9) were also found to be at increased risk of hr HPV infection. However HIV positive women on antiretroviral drugs were found to be at a reduced risk of hr HPV infection (OR: 0.4; 95% CI: 0.2-0.7) compared to HIV positive women not yet on treatment.

Table 5 shows the result of the multivariate analysis in three models to evaluate the relationship between HIV related variables and hr HPV status. Though HIV infection, CD4 count, high HIV viral load and the use of antiretroviral drugs were found to be associated with hr HPV infection status at the initial model, after adjusting for age only three variables of HIV positive status, CD4 count and use of antiretroviral drugs retained their independent association with hr HPV infection at the 2nd (adjusted for HIV status and treatment) and final models (in addition to the adjustment made in the initial and 2nd model, further adjustments were made for life time sexual partnership, type of community and marital status. In the final model, women infected with HIV infection (OR: 1.8; 95% CI: 1.4-2.2) and had CD4 cell count < 200 cells (OR: 2.4; 95% CI: 1.7- 5.9) were found to be at increased risk of hr HPV infection. While HIV positive women on antiretroviral therapy (OR: 0.4; 95% CI: 0.3-0.5) and with CD4 count of 500 cells and above (OR: 0.7; 95% CI: 0.5-0.8) were found to be at lower likelihood of having hr HPV infection compared with those not on treatment or with CD4 count less than 500 cells.

## Discussion

Among women enrolled in this study, the overall prevalence of hr HPV infection was 19.6% and the prevalence of multiple hr HPV infection was 5.4%. The prevalence of hr HPV infection in HIV positive women (24.5%) was significantly higher than the prevalence in HIV negative women (15.9%; OR: 1.7; 95% CI: 1.1-3.1). The most common hr HPV strains were 16 (3.9%), 35 (3.5%), 58 (3.3%) and 31(3.3%), however it was only HPV 16 (OR: 3.1; 95% CI: 1.3-6.3) and 35 (OR: 3.6; 95% CI: 1.4-6.9) genotypes that were found to have significant association with HIV infection. HIV positive women with a CD4 count of < 200 were found to be at higher risk of hr HPV infection than their counterpart with higher CD4 cell count (OR: 2.4; 95% CI: 1.7- 5.9). The use of antiretroviral drugs (OR: 0.4; 95% CI: 0.3-0.5) and CD4 cell count above 500 cells (OR: 0.7; 95% CI: 0.5-0.8) were found to be associated with a lower HPV prevalence than HIV positive women not on therapy or with CD4 count <500 cells respectively.

The overall hr HPV prevalence of 19.6% though similar to 18.3% reported from Ibadan, Nigeria [23], is higher than 14.6% reported from Irun, a rural and agrarian community in Osun state of Nigeria [24], suggesting a possible lower prevalence of hr HPV infection in rural communities. The present study further supports the reported rural–urban differences in HPV prevalence as the prevalence of hr HPV in rural communities (16.1%) was significantly lower than 23.9% in the urban settings. This finding is also confirmed by previous research in low-income countries, including Nigeria, that the prevalence of sexually transmitted infection is higher in urban than in rural areas [32,33]. This may be related to varying sexual behavior, lifetime sexual partnership and practices in the two settings or selection bias in various studies.

HPV 16, 35, 58 and 31 were the most common hr HPV genotype detected in this study, which is similar to findings reported by previous studies in our sub region [23,24,34]. However, the findings differed from the hr HPV distribution patterns described in other sub-Saharan African regions, Americas, Asia and Europe [2,3,9,21,23-25,27,28,33],[35]. While HPV 16 and 18 were the commonest genotype detected in Europe and North America [3,9,32], in East Africa HPV 52 and 16 were the most common [2,3,9,25,27]. In southern Africa, though the predominance of HPV 16 and 18 were retained, HPV 16 lost its position as the most common next to HPV 18 [2,27]. This confirms the assertion by Ngandwe et al. that in sub-Saharan Africa, other hr genotypes other than HPV 16 and 18 may play a more major role in evolution of cervical cancer than previously thought [35]. However this needs to be further confirmed in samples of women with invasive cervical cancer.

In the studied sample, the prevalence of hr HPV and multiple hr HPV infection were found to be significantly higher in HIV positive women (24.6% and 8.2%) than in HIV negative women (15.9% and 3.9%) respectively (OR: 1.7; 95% CI: 1.1-3.1, OR: 2.6; 1.3-5.3). However statistically significant association with HIV infection was noted only for hr genotypes HPV 35 and 16 after controlling for confounders (see Table 4). Women infected with HIV were 3.6 and 3.1 times likely to be infected with HPV 35 (OR: 3.6; 95% CI: 1.4-6.9) and 16 (OR: 3.1; 95% CI: 1.3-6.3) respectively than HIV negative women. The reported higher hr HPV prevalence in HIV infected women noted in this study were similar to previous observations from other regions of Africa, south America and Europe connoting that HIV infected women are at increased risk of hr HPV infection [2,3,13,26,27,34-37]. The observed increase in prevalence has been attributed to HIV related immunosuppression [6,32,38]. Strickler and colleagues confirmed in two separate studies an increased prevalence of HPV, persistence and decreased resolution of HPV infection in HIV positive women compared to HIV negative women [18,39]. Clifford and colleagues showed in their meta-analysis involving 20 publications that apart from viral types, immune-suppression of HIV infection significantly increased the risk for HPV infection and invasive cancer [38]. In addition, the reported more frequent multiple HPV types in HIV-positive women was consistent with previous reports [8-10], and may be attributable to the common mode of transmission of HPV and HIV; persistence of HPV as a result of the inability to clear HPV infections; as well as reactivation of latent HPV infections [38].

The higher prevalence of hr HPV infection in HIV positive women with CD4 count <200 cells/mm3 and significantly lower prevalence in those with CD4 count >500 cells found in this study further confirms the results of several studies that have consistently shown a higher prevalence of HPV infection and greater persistence of HPV infections in HIV positive women with CD4 < 200 cells/mm 3 [4,6,18,38,39]. The noted associations are reportedly due to severe immune-deficiency. In the presence of severe immunodeficiency the body’s capacity to suppress latent infection is impaired and the risk of HPV infection is increased [17,18,39,40]. HIV infection could also impact on the natural history of HPV by impairing the virus’s ability to escape the immune system [4,17,18,40].

In this study we were unable to confirm the earlier reported role of high HIV viral load on the prevalence and burden of hr HPV infection [4,10], as the initial association of hr HPV infection and viral load >10,000 copies (OR: 3.1; 95% CI: 1.1-9.9) was not retained after controlling for confounders in the 2nd and final models (see Table 5). The reported association in the other studies may be due to confounders of HIV treatment, CD4 cell count and sexual behaviour which were controlled for in this study [4,10,18,38].

A statistically significant association was found between hr HPV and use of antiretroviral drugs (ART) as HIV positive women on antiretroviral therapy were found to be at lower risk of acquiring hr HPV infection than those not on antiretroviral drugs (OR: 0.4; 95% CI: 0.3-0.5). This finding supports previous studies and also the hypothesis that ART has the potential to restore the immune response against HPV [40-44]. The use of ART may therefore reduce HPV persistence and reduce the occurrence of HPV infection and even favour regression [8,21,40].

The combined relatively low prevalence of HPV vaccine related HPV 16 and 18 reported in this study were comparable both in HIV positive and negative women. Also the combined prevalence in this study corroborate other findings reported from our sub-region [23,24,34], suggesting that the currently available vaccine based on HPV 16 and 18 will be useful in only a minority of women irrespective of their HIV status, as genotypes other than HPV 16 and 18 were found to constitute the majority of the circulating hr HPV genotypes.

Our results need to be interpreted with caution, as it was conducted in 2 states out of 36 states in Nigeria. However, Lagos as the commercial capital of Nigeria and former administrative capital is the sociocultural, behavioural and diverse ethnic melting point of the country. As such information obtained may be generalizable to the entire country. Secondly, being a cross-sectional study, it may have missed possible variations of specific HPV types overtime. However, as the study was conducted over a one year period this concern may have been taken care of. In addition, the recruitment of about 90% HIV positive group from the clinic and majority of HIV negative women from the community may have introduced some selection bias. This method of recruitment was used because of low HIV prevalence in Lagos and Ogun states. The HIV positive women in the HIV treatment Centre were used in this study because over 65% of them reside in the same setting as the women recruited in the community. Secondly if this strategy was not adopted, sufficient number of HIV positive women and those on antiretroviral therapy will not be recruited. In addition, there were no statistically significant differences in the sociodemographic characteristics between the two groups except in their HIV status. Mindful of the possible effect of this on the validity of our results, we controlled for the possible confounders in multivariate logistic regression using three models.

The major strength of this study is the recruitment of women of diverse characteristics including HIV positive and negative women from rural as well as urban areas, those on treatment and not on treatment, thus increasing the feasibility of generalizing the findings. It is also the first study, to the best of our knowledge, in our sub-region that evaluated the effect of HIV infection and treatment on hr HPV prevalence and distribution. In addition two sample size estimates were calculated (prevalence and comparison of proportion in two groups). This assured us that the study had sufficient statistical power to detect the expected effects.

## Conclusion

HPV 16, 35, 58 and 31 were the most common hr HPV infections in the population studied and HIV positive women are at higher risk of acquiring HPV infection. The current HPV vaccine prevents genotypes 16 and 18, which accounted for only a minority of hr HPV infection (21.7%) with no significant difference between HIV negative and positive women. The use of antiretroviral drugs was found to be protective against HPV infection. Although further studies with a larger number of participants and wider geographic spread are required in order to comprehensively characterize the HPV distribution in Nigerian women, this study raises important issues such as;

1) The effectiveness of the currently available HPV vaccine in our sub region.

2) The rate of HPV 35, 58 and 31 in invasive cancer cells in our setting.

3) The role of long term antiretroviral drugs on cervical cancer incidence, that should be further investigated to enable the development of a robust cervical cancer prevention and control strategy for all women in our sub region.

## Methods

### Study design

A cross sectional study among women of known HIV status enrolled into a study primarily to evaluate the utility of direct visual inspection of the cervix in diagnosing premalignant lesions of the cervix in HIV positive women.

### Study setting

The study was conducted at Nigerian Institute of Medical Research (NIMR) Lagos, cervical cancer screening Clinic and during community cervical cancer screening outreach programs in Lagos and Ogun States of Nigeria. These are two contiguous states in southwestern Nigeria, which together are inhabited by approximately 13 million people of whom 48.8% are women [45]. The cervical cancer screening outreach programs were conducted in 10 communities. Three urban (Surulere, Ikeja and Gbagada) and four rural (Mushin, Iju, Ikorodu and Egbeda) communities belong to Lagos State while one urban (Sagamu) and two rural (Ifo and Ibafo) are situated in Ogun state. The average distance from the communities to NIMR ranged from 2 to 70 kilometers. The population in the rural communities to a large extent is of low socioeconomic status and poorly educated in comparison to the urban population with relatively higher socioeconomic status and education. Within the urban communities however, pockets of slums exist, with living conditions similar to or worse than those in the rural areas.

NIMR is the apex medical research institution in Nigeria charged with the responsibility to conduct research into diseases of public health importance. The Institute currently provides comprehensive HIV care, treatment and support for over 20,000 patients of whom 62.9% are women. Over seventy-five percent of the patients come from Lagos and Ogun state (where majority of the HIV negative women in this study were recruited during community outreach programmes); with the remaining from the other neigbouring states and west African countries. All services at the Centre are provided free of charge.

### Study population

The study population was adult females aged 18 years and above seen at the cervical cancer screening clinic, Nigerian Institute of Medical Research Lagos and during community cervical cancer screening outreach programmes. While 80% of the HIV positive women were recruited from the cervical cancer screening clinic; the remaining were recruited during the community outreach. The HIV negatives were mainly recruited from the women who attended the various community outreaches cervical screening programme. However it is important to note that over 50% of the patient in our HIV treatment centre are from these same communities. The women were recruited after obtaining a written informed consent in both settings.

### Sample size determination

The sample size for this study was based on a reference prevalence value of hr HPV among women in a community based study conducted in Ibadan, Nigeria of 18.3% [21]. The study sample size was calculated according to the following formula: N = Zα^2^P (1-P)/d^2^, where Zα is the Z statistic for a 95% confidence level, N is the sample size, p is the prevalence of hr HPV, and d is the precision [46]. Based on this calculation, screening 229 women aged 18 years and above was considered sufficient for identifying women with hr HPV in each arm. To further ensure that the study is sufficiently powered to detect the effect of HIV treatment on hr HPV, a power calculation was further performed. Calculator from the EpiTools epidemiological calculators websites were used to generate power calculations:

http://epitools.ausvet.com.au.

http://epitools.ausvet.com.au/content.php?page=2Proportions&P1=0.15&P2=0.4&conf=0.95&Power=0.8&Ratio=1&Tails=2

These site calculators use standard assumptions based on normal approximation to the binomial distribution. The basic formula for comparing two proportions is: N = (Z_α_ + Z_β_) × ((p_1_)(1-p_1_) + (p_2_)(1-p_2_))/(p_1_-p_2_)^2^, where we used confidence interval of 95% for computation (Z_α_ = 1.96). Assuming sample sizes of 57 for each group and using an estimate of 40% in ARV drug naive group and 15% in ARV treatment group (difference of 25%), we arrived at 80% power to detect reduction in the rate of hr HPV in the antiretroviral drug group using a 2-tailed test.

### Data collection

Information on the socio-demographic, sexual and reproductive characteristics, HIV status and treatment history of women who consented to be part of the study were collected by trained female research assistants using a study record form designed for this study by OE, the Principal Investigator (PI). The laboratory information (HIV status, CD4 cell count, HIV plasma viral load and hr HPV genotypes) was extracted from the participant’s laboratory results and entered into the relevant portion of the study record form. The entered information was cross checked with the laboratory results by the PI to avoid entry error.

### Cervical sample collection

Trained Midwives and Doctors conducted the pelvic examination. Samples of cervical exfoliated cells from the ectocervix and endocervix were obtained using a cytobrush. The tip of the cytobrush, which contains cellular material was then placed into a transport medium and stored at 2-8°C immediately. All specimens were coded with the participants study number.

### Laboratory tests

#### HIV test

HIV test was conducted according to Nigerian National HIV testing and counseling guidelines in women with unconfirmed HIV status before enrolment into the study. Diagnosis was based on positive test on double ELISA based algorithm. However before initiation of antiretroviral therapy the initial test was reconfirmed using Western Blot.

#### Viral load and CD4 cell count test

The tests were conducted at the Human Virology Laboratory, NIMR. Whole blood of the HIV positive women were used to perform CD4 assay using the Cyflow Counter and Kits (Partec, Germany) according to the Manufacturer’s instructions. The viral load assay was performed using Roche Amplicor HIV-1 monitor test (version 1.5) according to manufacturer’s instruction.

#### DNA extraction and detection

All samples were subjected to centrifugation at 200 rpm for 10 minutes. Precipitated cells were digested using digestion buffer containing protein kinase. HPV DNA was extracted with phenol- chloroform, re-suspended in 100 μl elution buffer and stored at −20°C prior to molecular analysis. Quality and integrity of DNA sample for PCR was verified by amplification of a 268 base pair region of human B-globin gene. Specimens with negative internal control amplification were excluded. All the B-globin positive samples were amplified with HPV4A ACE PC (Inqaba, South Africa). To detect the genotype of the 13 high risk HPV strains, samples were amplified using the specific primers HPV 16, 18, 31, 33, 35, 39, 45, 51, 52, 56, 58, 59, 68.

### Data analysis

Information entered into the study record forms were coded and analyzed using the SPSS version 19.0 (SPSS Inc. Chicago, IL) statistical packages. The main outcome variable was hr HPV positivity, defined as presence of any of the 13 virus strains mentioned above. First level analysis was to determine the overall prevalence of hr HPV (defined as presence of any of the 13 hr HPV strains) among the women and the prevalence of specific hr HPV strains by HIV status and adjusted for age, treatment history and life time sexual partnership. Since the hr HPV prevalence was not very high, we used multivariate model to estimate the odds ratio for independent variables associated with hr HPV infection. Bivariate analysis was first performed to assess the association of variables with hr HPV infection. All factors independently associated with hr HPV infection in the bivariate analysis were considered as confounders and introduced in a step wise manner in the models. In the first step, we started with the crude association between the variables and main outcome adjusted by age (Initial Model), then we adjusted for HIV status and treatment (Model 2), and finally in a step wise manner introduced the variables that were shown to be statistically associated with hr HPV infection in the bivariate analysis (Final Model). The results were reported in percentages, Odds ratios (OR) or adjusted ORs and their 95% confidence intervals (CI).

### Ethical considerations

Approval for the study was obtained from the Institutional Review Board, Nigerian Institute of Medical Research, Lagos Nigeria. A written informed consent was obtained from the women invited to be part of the study after detailed information about the study. Impartial witnesses who were not members of NIMR staff assisted the consenting process for the low literates.

### Definition of variables

•HIV treatment status: The use of antiretroviral therapy among the HIV positive women for more than 3 months.

•Viral load: The level of HIV RNA copies/mL of plasma measured by Roche Ampliclor HIV-1 monitor test (version 1.5).

•CD4 Cell count: The level of body’s immunity measured as the number of CD4 T lymphocytes per mm3 of blood by Cyflow Counter and Kits.

•High risk Human Papilloma Virus (hr HPV): The 13 oncogenic HPV strains of 16, 18, 31, 33, 35, 39, 45, 51, 56, 58, 59 and 68.

## Competing interests

The authors declare that they have no competing interests.

## Authors’ contributions

All the authors participated in the planning and design of the study, and all read and approved the final manuscript. EO conceived the study, recruited the women, performed the statistical analysis and produced the first draft. In collaboration with NF and IAOU, OE defined the protocol for molecular analysis, while NF performed the molecular laboratory studies. PO and KOP reviewed the data analytic plan, the statistical output for accuracy and appropriateness and reviewed all the draft manuscripts for important intellectual content. All authors read and approved the final manuscript.
